# Effects of the traditional Chinese medicine *Yi Shen Jian Gu* granules on aromatase inhibitor-associated musculoskeletal symptoms: a study protocol for a multicenter, randomized, controlled clinical trial

**DOI:** 10.1186/1745-6215-15-171

**Published:** 2014-05-15

**Authors:** Nan Peng, Yi Zhang, Cong Ma, Ming-Wei Yu, Guo-Wang Yang, Qi Fu, Wei-Ru Xu, Xiao-Min Wang

**Affiliations:** 1 Oncology Department, Beijing Hospital of Traditional Chinese Medicine affiliated with Capital Medical University, No 23, Back Road of Art Gallery, Dong Cheng District, Beijing 100010, China

**Keywords:** Traditional Chinese medicine, Aromatase inhibitor-associated musculoskeletal symptoms, Breast cancer

## Abstract

**Background:**

Aromatase inhibitors (AIs) are widely used as an adjuvant endocrine treatment in postmenopausal women with early-stage breast cancer. One of the main adverse effects of AIs is musculoskeletal symptoms, which leads to a lower quality of life and poor adherence to AI treatment. To date, no effective management of aromatase inhibitor-associated musculoskeletal symptoms (AIMSS) has been developed.

**Methods/design:**

To determine whether the traditional Chinese medicine *Yi Shen Jian Gu* granules could effectively manage AIMSS we will conduct a multicenter, randomized, double-blind, placebo-controlled clinical trial. Patients experiencing musculoskeletal symptoms after taking AIs will be enrolled and treated with traditional Chinese medicine or placebo for 12 weeks. The primary outcome measures include Brief Pain Inventory-Short Form, Western Ontario and McMaster Universities Osteoarthritis Index, and Modified Score for the Assessment and Quantification of Chronic Rheumatoid Affections of the Hands, which will be obtained at baseline and at 4, 8, 12 and 24 weeks.

**Discussion:**

The results of this study will provide a new strategy to help relieve AIMSS.

**Trial registration:**

ISCTN: ISRCTN06129599 (assigned 14 August 2013).

## Background

Third-generation aromatase inhibitors (AIs; that is, anastrozole, letrozole and exemestane) have become the standard of care for the adjuvant treatment of hormone-responsive breast cancer in postmenopausal women. For many years, tamoxifen was the cornerstone of endocrine therapy [1]. However, recent clinical trials of AIs have shown benefits over tamoxifen. The main advantages are improvement of disease-free survival, decreased rates of contralateral breast cancer and a more favorable toxicity profile, with lower rates of thromboembolic phenomena and endometrial malignancy [2-8]. These trials reported incidences of musculoskeletal symptoms ranging from 5% to 35%. Observational studies, however, have shown that aromatase inhibitor-associated musculoskeletal symptoms (AIMSS) are more prevalent than originally reported [9,10]. In a cross-sectional survey of 200 women receiving adjuvant AI therapy for breast cancer, 94 (47%) reported AI-associated joint pain, and 88 (44%) reported joint stiffness; nearly 13% of patients discontinued therapy because of intolerable musculoskeletal symptoms [9]. Because the mechanisms mediating AIMSS are not clearly understood, effective management of AIMSS has not yet been developed.

Potential interventions for AIMSS usually include nonsteroidal anti-inflammatory drugs, analgesics, calcitriol agents, vitamin D, and exercise. Some patients even have to switch AIs or switch to tamoxifen because of severe musculoskeletal symptoms [11,12]. However, it is not clear whether any of these interventions have had a dramatic effect on musculoskeletal symptoms. Therefore, due to the unsatisfactory response to current symptomatic treatments, many patients seek help from traditional Chinese medicine (TCM).

TCM, which has a unique advantage in alleviating symptoms and improving quality of life, has been widely applied in China for the treatment of cancer-related side effects, such as nausea, vomiting, fatigue, tidal fever, and pain [13,14]. Postmenopausal women with AIMSS exhibit a specific pathological change associated with deficiency of kidney essence and depression of liver qi, according to TCM theory [15]. Insufficient kidney essence results in failure to nourish the related body constituents and organs, including bone and joints. Liver qi depression leads to stagnation of qi movement, manifested as pain [16]. Through prescription of *Yi Shen Jian Gu* granules (YSJG), which, according to therapeutic principle of TCM, would tonify the kidney, fortify the bone, soothe the liver, regulate qi and unblock the collateral, we noted that AIMSS could be partly relieved in clinical practice. YSJG has been commonly used in our hospital for the treatment of musculoskeletal symptoms, including bone pain, myalgia, and arthralgia, in patients with osteoporosis or arthrosis. However, useful empirical observation is insufficient. Therefore, there is a need for a well-designed clinical trial to investigate the efficacy and safety of YSJG on AIMSS. The results of this study will provide clinical evidence on the efficacy and safety of YSJG in patients with AIMSS.

## Methods/design

### Study design

**Figure 1:**
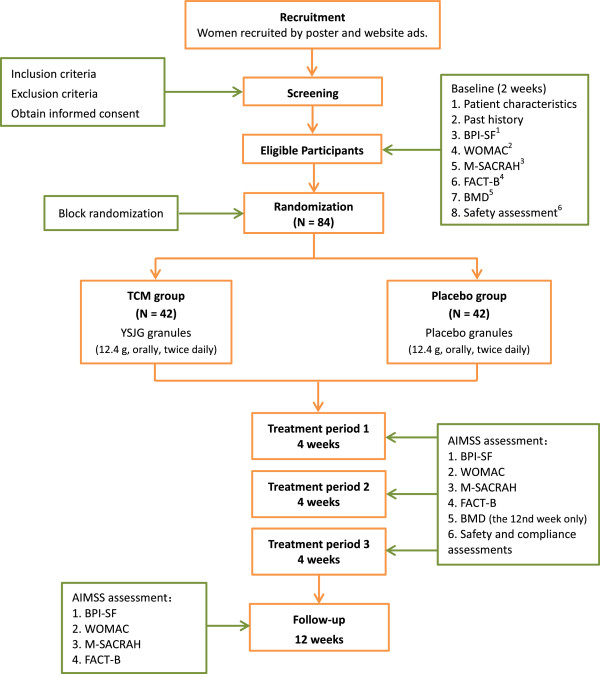
**Project overview. **^1^BPI-SF: Brief Pain Inventory-Short Form (Chinese version). ^2^WOMAC 3.1 Index: Western Ontario and McMaster Universities Osteoarthritis Index (Chinese version). ^3^M-SACRAH: Modified Score for the Assessment and Quantification of Chronic Rheumatoid Affections of the Hands (Chinese version). ^4^FACT-B: the Functional Assessment of Cancer Therapy breast cancer-specific quality of life tool (Chinese version). ^5^BMD: bone mineral density. ^6^Safety assessment: blood routine test, urine routine test, feces routine test, kidney function test, liver function test, estradiol (E2), follicle-stimulating hormone, tumor markers (CEA, CA125, and CA153). AIMSS, aromatase inhibitor-associated musculoskeletal symptoms; TCM, traditional Chinese medicine; YSJG, *Yi Shen Jian Gu* granules.

This study is a multicenter, randomized, double-blind, placebo-controlled clinical trial with two parallel arms (Figure 1). The aim of the study is to evaluate whether TCM (YSJG) is effective to relieve AIMSS in patients with breast cancer. Eighty-four participants will be recruited from three centers: Beijing Hospital of Traditional Chinese Medicine affiliated with Capital Medical University, Beijing Cancer Hospital, and Guang’ Anmen Hospital affiliated with China Academy of Chinese Medical Sciences. All participants will be prescribed one of the following AIs: anastrozole (Arimidex, AstraZeneca Pharmaceutical Co., 587 Old Baltimore Pike, Newark, Delaware, 19702, USA; 1 mg/day), letrozole (Femara, Novartis Pharma Schweiz AG, Schaffhauserstrasse 4332 Stein, Switzerland, or Letrozole Tablets, Jiang Su Heng Rui Medicine Co., Ltd, No 38, the Yellow River Road, Lianyungang City, Jiangsu Province; 2.5 mg/day), or exemestane (Aromasin, Pfizer Inc., Localita Marino del Tronto 63100, Italy; 25 mg/day). After a 2-week run-in period, eligible participants will be randomly assigned to the TCM group or the placebo group. Considering that the majority of postmenopausal women who are taking AI need to receive calcium and vitamin D, and in order to maintain the homogeneity between patients, we will prescribe calcium carbonate and vitamin D3 tablets for all participants (each tablet is comprised of 600 mg calcium and 125 IU vitamin D3; participants take two tablets daily for 12 weeks; Wyeth Pharmaceutical Co. Ltd, No 4, Baodai West Road, Suzhou City, Jiangsu Province). The TCM group will receive YSJG granules, and the placebo group will receive placebo granules. Both groups will have a 12-week treatment period and a 12-week follow-up period. Five visits will be scheduled for each patient in their respective centers: one visit each at weeks 0, 4, 8, 12, and 24.

### Ethical issues

The trial will be conducted in accordance with the Declaration of Helsinki and the Ethical Guidelines for Clinical Research, and the trial protocol has been approved by the Research Ethical Committee of Beijing Hospital of Traditional Chinese Medicine Affiliated with Capital Medical University (ref: 201337). This trial was registered with ISRCTN at Current Controlled Trials (ISRCTN06129599).

### Study participants

Participants will be recruited from three hospitals (Beijing Hospital of Traditional Chinese Medicine, Beijing Cancer Hospital, and Guang’ Anmen Hospital) by poster and website advertisements. Informed consent will be obtained from all participants before randomization. Eligibility and exclusion criteria are presented in Table 1.

**Table 1:** Eligibility criteria

	**Inclusion criteria**
1.	Pathologically confirmed stage I to III breast cancer with no recurrence and metastasis.
2.	Completed chemotherapy and/or radiotherapy.
3.	Currently using a third-generation aromatase inhibitor (anastrozole, letrozole, or exemestane) and self-reported ongoing musculoskeletal symptoms (arthralgia and/or stiffness and/or swelling in one or more joints, bone pain, myalgia, carpal tunnel syndrome, trigger finger), which started or worsened after initiation of aromatase inhibitor therapy and has continued for more than 1 month, and who had a baseline worst pain score over the past week on the Brief Pain Inventory-Short Form ≥3 points on a scale of 0 to 10.
4.	Eastern Cooperative Oncology Group performance status 0-2.
5.	Provided written informed consent before enrollment.
	**Exclusion criteria**
1.	Patients with endocrine and any other diseases influencing bone metabolism (for example, hyperthyroidism, hypothyroidism, diabetes, Cushing’s syndrome, chronic liver disease, nephropathy, myeloma, bone tumor, or bone metastasis).
2.	Used of agents influencing bone metabolism (for example, glucocorticoid, thyroid hormone, heparin, anticonvulsants, diuretics, or bisphosphonates), except calcium agents, within the past 3 months.
3.	Contraindications to calcium agents and vitamin D.
4.	Diagnosed with primary osteoarticular diseases.
5.	Presence of other primary tumors and severe heart, liver, kidney, and hematopoietic system diseases.
6.	Presence of pregnancy, mental illness, or cognitive handicap.

### Interventions

For the treatment group, YSJG granules are composed of 12 herbs, including *Radix rehmanniae Preparata* (ShuDiHuang), *Semen cuscutae* (TuSiZi), *Rhizoma cyperi* (XiangFu), *Rhizoma chuanxiong* (ChuanXiong), *Rhizoma corydalis* (YanHuSuo), *Caulis trachelospermi* (LuoShiTeng), and so forth. Because the formula for YSJG is currently being patented, the ingredients in YSJG cannot be published at this time.

For the control group, placebo granules are made from dextrin (95%) and *Herba pogostemonis* (5%) in order to achieve the same color, smell, taste, and texture as that of YSJG granules.

Both YSJG and placebo granules were manufactured by Beijing Tcmages Pharmaceutical Co. Ltd (No 5, Niu Hui Street, Shun Yi District, Beijing) according to the standards of Good Manufactoring Practice. Patients will be instructed to dissolve a sachet of granules (12.4 g) in 200 mL hot water and to take the solution orally twice a day for 12 weeks.

### Randomization and blinding

Block randomization was carried out in a 1:1 ratio according to the sequence generated with SAS (Version 7.0; Channel-leadian pharmaceuticals R&D, Co., Ltd; No 19, Xiao Ying Road, Chao Yang District, Beijing). Drug codes, numbered from 1 to 84, and contained group assignments will be generated according to randomization numbers. Eligible patients will be randomized into the YSJG group or the placebo group by obtaining medicines associated with the given drug codes in accordance with the order of visits. Group assignments will not be revealed until the entire study is completed. Participants, investigators, statisticians, and all study staff are blinded.

### Outcome measures

#### Primary outcome measure

As pain is the most common complaint of AIMSS, we selected the Brief Pain Inventory-Short Form (BPI-SF) as the primary outcome measure to evaluate general pain. The BPI-SF, created by the Pain Research Group, is a 14-item questionnaire to measure both the intensity of pain as well as the interference of pain in the patient’s life on a scale ranging from 0 to 10.

#### Secondary outcome measures

##### Quality of life instruments

The Western Ontario and McMaster Universities Osteoarthritis Index (WOMAC) is a patient-reported outcome measure for assessing osteoarthritis of the knees or hips. It consists of 24 questions (5 pain, 2 stiffness, and 17 physical function) and can be completed in less than 5 minutes. We selected the WOMAC index to assess joint pain, stiffness, and functional status in the knees.

The Modified Score for the Assessment and Quantification of Chronic Rheumatoid Affections of the Hands (M-SACRAH) consists of three domains assessing pain, stiffness, and functional status in patients suffering from hand osteoarthritis and rheumatoid arthritis, answered on 100-mm visual analog scales. We selected the M-SACRAH to assess joint pain, stiffness, and functional status in the hands.

The Functional Assessment of Cancer Therapy-Breast (FACT-B), a quality of life instrument to assess the well-being of cancer patients, measures physical, social/family, emotional, and functional well-being as well as additional concerns commonly reported in breast cancer patients. The FACT-B scale has five response levels (ranging from ‘not at all’ to ‘very much’). Changes in the scores on these scales before and after intervention will be compared between the TCM group and the placebo group.

##### Bone mineral density

Musculoskeletal symptoms and bone loss are identified as the two main adverse effects of AIs. One study reported a correlation between AIMSS and osteoporosis (bone fractures) [17]. In order to investigate the effects of YSJG on bone loss, bone mineral density (BMD) of the L2-L4 region of the spine and hip will be assessed before and after treatment (at weeks 0 and 12).

### Safety assessments

In order to assess the safety of YSJG, we will perform the following tests on all participants at the screening phase (week 0) and after treatment (week 12): blood routine test, urine routine test, feces routine test, kidney function test, liver function test, estradiol (E2), follicle-stimulating hormone, and serum levels of tumor markers (CEA, CA125, and CA153). In addition, investigators will ask every subject at each visit whether they have experienced any adverse events (AEs) during the study period. If there are any AEs, the investigator will provide appropriate treatment to the subject immediately and record AEs in a dedicated document in the case report form, including its severity and causality with the experimental agent. In the case of serious adverse events (SAEs), the investigator will offer appropriate treatment to the subject immediately and report the event to the Institutional Review Board within 24 hours from the time of recognition. If necessary, blinding will be broken by adequate procedure, and the documented procedure will be kept in the investigator study file.

### Sample size calculation

As there has been no similar TCM research on AIMSS to date, we calculated sample size according to another AIMSS study that used the same primary outcome measure (BPI-SF) [18]. From the results of this previous study, the treatment group had a reduction of 1.5 points (the standard deviation was 2.1) on the mean BPI-SF worst pain compared with the control group. Therefore, 34 participants per group were deemed sufficient to achieve 90% power and a (one-sided) 5% significance level in detecting treatment differences. Considering a total withdrawal and dropout rate of 20%, we concluded that a total of 84 patients (42 per arm) would need to be recruited to ensure statistically significant results.

### Statistical analysis

Efficacy and safety analyses will be conducted according to the intention-to-treat principle. Missing values will be imputed by the last-observation-carried-forward method. All statistical analyses will be performed using Statistical Packages of Social Sciences software (SPSS 18.0, Channel-leadian pharmaceuticals R&D, Co., Ltd; No 19, Xiao Ying Road, Chao Yang District, Beijing). Statistical significance will be defined as a one-sided *P*-value of <0.05. Demographic, clinical, and outcome variables will be described using means and standard deviations for continuous variables and percentages for categorical variables. The primary analysis will be to compare the mean pain scores from BPI-SF, the WOMAC, and the M-SACRAH and the mean joint stiffness and function scores from the WOMAC and the M-SACRAH between the TCM group and the placebo group. The secondary analysis will be to compare the mean scores from FACT-B and changes in BMD before and after treatment. Student’s t-tests and repeated-measure analyses of variance with a time-interaction term will be used to compare group differences.

### Data collection and monitoring

This is a 24-week clinical trial, in which participants need to take research medication for 12 weeks, accept a 12-week follow-up, attend five assessment visits, obtain two sets of laboratory tests (safety assessments) and BMD analysis, fill in four sets of questionnaires at each visit, and stop taking other herbal medications (Table 2).

**Table 2:** Data collection schedule

	**Screening**	**Treatment**			**Follow-up**
Visit number	1	2	3	4	5
Week number	−2 to 0	4	8	12	24
Patient characteristics	✘				
Past history	✘				
AIMSS	✘				
ECOG	✘				
BPI-SF	✘	✘	✘	✘	✘
WOMAC	✘	✘	✘	✘	✘
M-SACRAH	✘	✘	✘	✘	✘
FACT-B	✘	✘	✘	✘	✘
Bone mineral density	✘			✘	
Safety assessment	✘			✘	
Combined medication	✘	✘	✘	✘	✘
Adverse events		✘	✘	✘	✘
Compliance		✘	✘	✘	

AIMSS, aromatase inhibitor-associated musculoskeletal symptoms; BPI-SF, Brief Pain Inventory-Short Form; ECOG, Eastern Cooperative Oncology Group; FACT-B, The Functional Assessment of Cancer Therapy-Breast; M-SACRAH, Modified Score for the Assessment and Quantification of Chronic Rheumatoid Affections of the Hands; WOMAC, Western Ontario and McMaster Universities Osteoarthritis Index.

Beijing Qihuang Medicine Clinical Research Center is responsible for quality control. Censors have regular visits (once a month) to monitor protocol violations, the recruitment rate, AEs and participant compliance.

## Discussion

Up to half of patients on AI therapy experience musculoskeletal symptoms, and up to 20% will discontinue therapy because of intolerable side effects; therefore, AIMSS has become an important clinical issue requiring effective management.

Effective management of AIMSS is still a mystery in clinical practice, as the mechanisms behind AIMSS are not clearly understood. A number of small interventional trials investigating acupuncture [19,20], vitamin D [18], glucosamine [21], short-term low-dose prednisolone [22], thymosin α1 [23], duloxetine [24] and yoga [25] have provided various treatment strategies. However, most of these trials have had some methodological and practical limitations, including small sample sizes, larger-than-anticipated drop-out, a single-center design, lack of control group and blinding, and a short follow-up period. Therefore, developing appropriate management strategies for AIMSS will require further investigation. To date, there is still a lack of prospective, randomized, controlled trials to provide powerful evidence supporting the use of various treatment modalities. Our study will be the first randomized, double-blind, placebo-controlled clinical trial analyzing the efficacy and safety of TCM in patients with AIMSS.

There are several limitations to our study. One limitation is the absence of specific measures for AIMSS. AIMSS is a set of symptoms involving the bone, muscles and joints. There are no questionnaires specially designed for AIMSS. Through reviewing the literature and reported studies [20,23,26], rheumatological questionnaires may be validated in the longitudinal assessment of joint symptoms, although the pathological processes are likely to be different. We selected WOMAC and M-SACRAH that focus on joint pain, stiffness, and functional status in the hands and knees, which are the most common locations of pain. Another limitation is the indeterminate diagnostic criteria of AIMSS. Musculoskeletal symptoms are reported according to different terms (for example, arthralgia, joint symptoms, joint pain, and musculoskeletal symptoms) in the literature. Because of the lack of clear diagnostic criteria, various clinical trials have reported a wide range of AIMSS incidence and research objects, which confuses physicians and investigators and makes it challenging to recognize the manifestations of AIMSS and to establish acceptable inclusion criteria. In order to clear the clinical characteristics of AIMSS and inclusion criteria, we defined AIMSS, through reviewing the literature [10,11,27,28], as self-reported ongoing musculoskeletal symptoms, including arthralgia, joint stiffness, joint swelling, bone pain, myalgia, carpal tunnel syndrome, and trigger finger, which started or worsened after taking an AI.

The results of this study will provide a new evidence-based treatment strategy for patients suffering from AIMSS.

## Trial status

The trial is currently enrolling participants.

## Abbreviations

AE: Adverse event; AI: Aromatase inhibitor; AIMSS: Aromatase inhibitor-associated musculoskeletal symptoms; BMD: Bone mineral density; BPI-SF: Brief Pain Inventory-Short Form; ECOG: Eastern Cooperative Oncology Group; FACT-B: The Functional Assessment of Cancer Therapy-Breast; M-SACRAH: Modified Score for the Assessment and Quantification of Chronic Rheumatoid Affections of the Hands; TCM: Traditional Chinese medicine; WOMAC: Western Ontario and McMaster Universities Osteoarthritis Index; YSJG: *Yi Shen Jian Gu* granules.

## Competing interests

The authors declare that they have no competing interests.

## Authors’ contributions

All authors participated in the design of the study and performed the trial. NP drafted the manuscript. XMW, GWY, and MWY supervised and coordinated the clinical trial. MWY participated in statistical design. QF, WRX, YZ, CM and NP are responsible for recruiting the participants. All authors read and approved the final manuscript.
